# Characterizing Acupuncture *De Qi* in Mild Cognitive Impairment: Relations with Small-World Efficiency of Functional Brain Networks

**DOI:** 10.1155/2013/304804

**Published:** 2013-07-11

**Authors:** Lijun Bai, Ming Zhang, Shangjie Chen, Lin Ai, Maosheng Xu, Dan Wang, Fei Wang, Lihua Liu, Fang Wang, Lixing Lao

**Affiliations:** ^1^The Key Laboratory of Biomedical Information Engineering, Ministry of Education, Department of Biomedical Engineering, School of Life Science and Technology, Xi'an Jiaotong University, Xi'an 710049, China; ^2^Department of Radiology, The First Affiliated Hospital of Medical College, Xi'an Jiaotong University, Xi'an 710061, China; ^3^Baoan Hospital, Southern Medical University, Shenzhen 518101, China; ^4^Department of Nuclear Medicine, Beijing Tiantan Hospital, Capital Medical University, Beijing 100050, China; ^5^Center for Integrative Medicine, School of Medicine, University of Maryland, 520 W. Lombard Street, Baltimore, MD 21201, USA

## Abstract

As an intermediate state between normal aging and dementia, mild cognitive impairment (MCI) became a hot topic and early treatments can improve disease prognosis. Acupuncture is shown to have possible effect in improving its cognitive defect. However, the underlying neural mechanism of acupuncture and relations between *De Qi* and different needling depths are still elusive. The present study aimed to explore how acupuncture can exert effect on the reorganization of MCI and to what extent needling depths, associating with *De Qi* sensations, can influence the acupuncture effects for MCI treatment. Our results presented that MCI patients exhibited losses of small-world attributes indicated by longer characteristic path lengths and larger clustering coefficients, compared with healthy controls. In addition, acupuncture with deep needling can induce much stronger and a wide range of *De Qi* sensations both in intensity and prevalence. Acupuncture with deep needling showed modulatory effect to compensate the losses of small-world attributes existed in MCI patients while acupuncture with superficial needling did not. Furthermore, acupuncture with deep needling enhanced the nodal centrality primarily in the abnormal regions of MCI including the hippocampus, postcentral cortex as well as anterior cingulate cortex. This study provides evidence to understand neural mechanism underlying acupuncture and the key role of *De Qi* for MCI treatment.

## 1. Introduction

Alzheimer's disease (AD) accounts for 50–60% of all dementia [1], with incidence rates doubling every 5 years after the ages of 65. It is estimated that half of the population above 80 years old may have symptomatic AD and that this number will grow rapidly as life expectancy increases. As a prodromal stage of AD, mild cognitive impairment (MCI) refers to the clinical condition between the normal aging and AD.

MCI patients usually experience memory loss to a greater extent than one would expect for age, while they do not meet the criteria for AD [2]. However, it is reported that MCI has a high risk for AD progression and nearly 10–15% of them will convert to AD [3]. Early treatment is preferred to reduce burdens of patients' families, since the psychological and financial cost of AD is tremendous and rapidly rising. Patients often seek help through acupuncture hoping that such treatments might produce improvements in quality of life and delay cognitive decline [4, 5]. However, the underlying neural mechanism of acupuncture for MCI is still elusive. It is thus necessary and urgent to find out the modulatory effect of acupuncture on the treatment of MCI, which may provide opportunities for relatively early intervention of AD. 

One research investigated the effect of acupuncture on cognitive performance of multi-infarct dementia (MID) rats by using neuroethology measurements [6]. They found that acupuncture exerted a protective effect on cognitive impairment caused by cerebral multi-infarction in rats, and acupuncture has a specificity of cure. In addition, another randomized clinical trials have reported that electroacupuncture can exert favourable effects on activities of daily living, compared with drug therapy [7, 8]. 

Acupuncture improves cognitive function measured with MMSE compared with preintervention in eight patients with mild or moderate AD after 1 month of treatment [4]. However, most of previous studies have primarily focused on the relation between the acupuncture effects and the cognitive levels just by behavior measurements. One recent study clarifies the mechanisms of acupuncture in treating MCI and AD by using functional magnetic resonance imaging (fMRI). They found that, compared with the healthy control, acupuncture at Tai Chong (Liv3) and He Gu (LI4) can activate certain cognitive-related regions in both AD and MCI patients [9]. Considering that both AD and MCI are due to disconnections among several regions in a wide range of brain network, exploring the interregional connectivity within the whole brain networks, can further understand the modulated effect of acupuncture treatment for MCI.

According to the traditional Chinese medicine (TCM), acupuncture stimulation generally elicits *De Qi*, a composite of unique sensations interpreted as the flow of *qi *or “energy,” which is essential for clinical efficacy. Exploring such key component from modern biomedical viewpoint is necessary for understanding the specific mechanism underlying acupuncture intervention for MCI. In the present study, we aimed to explore the relations between *De Qi *sensations induced by different needling depths of acupuncture and their differential modulated effects on the reorganizations of whole-brain networks using nonrepeated event-related fMRI techniques. 

## 2. Methods

### 2.1. Subjects

Twelve aMCI patients were enrolled from the rehabilitation department of the Bao'an People's Hospital of Shenzhen (subjects' demographics shown in Table 1). MCI patients were diagnosed by a qualified neurologist using criteria for amnestic MCI [10], with mini-mental state examination (MMSE) scores >25 [11] and clinical dementia Rating (CDR) scale scores of 0.5 [12]. Twelve age-matched normal elderly were randomly selected from a community investigation of epidemiological research. All subjects were right handed according to the Edinburgh Handedness Inventory [13] and acupuncture naïve. Subjects were excluded if they had any significant medical, neurological, or psychiatric illness, or if they were taking medication or other substances known to influence cerebral function. After given a complete description of the study, all subjects signed the informed consent form. All protocols were approved by an ethic committee on human studies. 

### 2.2. Experimental Paradigm

**Figure 1:**
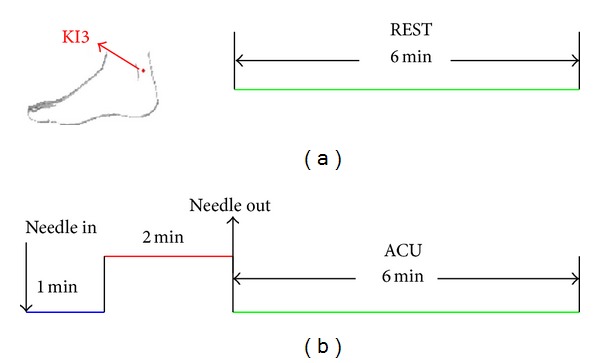
Experimental paradigm. (a) The paradigm for a resting state (REST) run lasting for 6 minutes. (b) The paradigm for acupuncture (ACU) for both DA and SA runs totally lasting for 9 minutes.

In this study, we adopted a novel experimental paradigm, namely, the NRER fMRI design to investigate the sustained effects induced by acupuncture administration [14]. For each group, the experiment consisted of two functional runs. At the beginning, a resting state (REST) scan was conducted for 6 minutes without any stimulation (Figure 1(a)). Then, the NRER experimental paradigm was conducted. Acupuncture was performed at acupoint KI3 on the right leg (Taixi, located on the medial border of the foot posterior to the medial malleolus, in the depression between the tip of the medial malleolus and Achilles tendon). This is one of the most frequently used acupoints and proved to have various efficacies in the treatments of dementia [15]. The needle was inserted vertically to a depth of 1-2 cm with deep needling (DA), but of 1-2 mm in superficial needling (SA). For both acupuncture DA and SA, we employed the NRER-fMRI design paradigm, incorporating 2 min needle manipulation, preceded by 1 min rest epoch and followed by 6 min rest (without acupuncture manipulation) scanning. All participants were not informed of the order in which these three runs would be performed and were asked to remain relaxed without engaging in any mental tasks. To facilitate blinding, they were also instructed to keep their eyes closed to prevent them from actually observing the procedures. According to participants' reports after the scanning, they affirmed keeping awake during the whole process. The presentation sequence of these three runs was randomized and balanced throughout the population, and every participant performed only one run in each day in order to eliminate potential long-lasting effect following acupuncture administration.

Acupuncture stimulation was delivered using a sterile disposable 38 gauge stainless steel acupuncture needle, 0.2 mm in diameter and 40 mm in length. Acupuncture administration was delivered by a balanced “tonifying and reducing [sic]” technique [16]. Stimulation consisted of rotating the needle clockwise and counterclockwise for 1 min at a rate of 60 times per min. The procedure was performed by the same experienced and licensed acupuncturist on all participants. As a concurrent psychophysical analysis, we used a verbal analog scale to ask participants to quantify the subjective sensations of acupuncture or deqi at the end of the acupuncture DA and SA runs. The sensations are all listed on the MGH acupuncture sensation scale (MASS), including aching, soreness, pressure, heaviness, fullness, warmth, coolness, numbness, tingling, throbbing, dull or sharp pain, and one blank row for subjects to add their own word(s) if the above descriptors did not embody the sensations they experienced during stimulation [17, 18]. The intensity of each sensation was measured on a scale from 0 to 10 (0 = no sensation, 1–3 = mild, 4–6 = moderate, 7-8 = strong, 9 = severe, and 10 = unbearable sensation). Since sharp pain was regarded to result from an inadvertent noxious stimulation rather than acupuncture deqi [19], we excluded the subjects for further analysis if they experienced sharp pain (greater than the mean by more than two standard deviations). In this cohort, none of subjects experienced the sharp pain. No subject opted to add an additional descriptor in the blank row provided.

### 2.3. Data Acquisition and Preprocessing

Magnetic resonance imaging data were acquired using a Tesla Signa (GE) MR scanner. Head movements were prevented by a custom-built head holder. The images were parallel to the AC-PC line and covered the whole brain. Thirty axial slices were obtained using a T2*-weighted single-shot, gradient-recalled echo planar imaging sequence (FOV = 220 mm × 220 mm, matrix = 64 × 64, thickness = 4 mm, TR = 2000 ms, TE = 30 ms, and flip angle = 77°). After the functional run, high-resolution structural information on each subject was also acquired using 3D MRI sequences with a voxel size of 1 mm^3^ for anatomical localization (TR = 2.1 s, TE = 4.6 ms, matrix = 256× 256, FOV = 230 mm × 230 mm, flip angle = 8°, and slice thickness = 1 mm).

For rest run, the data were preprocessed by removing the first 5 time points to eliminate nonequilibrium effects of magnetization. For acupuncture DA and SA, the postacupuncture resting state was adopted as the data sets for further analysis. All of data preprocessing procedures were conducted with the Statistical Parametric Mapping 5 (SPM5) (http://www.fil.ion.ucl.ac.uk/spm/). The images were corrected for the acquisition delay between slices, aligned to the first image of each session for motion correction, and spatially normalized to standard MNI template in SPM5 [20]. No subjects had head motions exceeding 1 mm movement or 1° rotation in any direction. The image data was further processed with spatial normalization based on the MNI space, resampled at 2 mm × 2 mm × 2 mm, and finally spatially smoothed with a 6 mm full-width-at-half maximum (FWHM) Gaussian kernel. The functional images were normalized to the Talairach stereotactic system. Finally, A band-pass filter (0.01 Hz < *f* < 0.08 Hz) was applied to remove physiological and high-frequency noise [21].

### 2.4. Anatomical Parcellation

After preprocessing, the fMRI data were segmented into 90 regions (45 for each hemisphere), using an anatomically labeled template [22] that has been widely used in previous neuroimaging studies via graph theoretical approaches [23–25]. For each subject, the representative time series of each region was estimated simply by averaging the fMRI time series over all voxels in the region.

### 2.5. Graph Construction

We calculated partial correlations between each pair of brain regions to reduce the indirect dependencies by other brain regions and to obtain a partial correlation matrix *R* [24]. Then, Fisher's transform was adopted to improve the normality of the partial correlation coefficients. Finally, a threshold (*r*) was related with the partial correlation coefficient (*R*_*ij*_) to convert *R* to a binary graph. In this step, we set any *R*_*ij*_ whose absolute value was greater than *r* to 1 and others to 0. And a false discovery rate (FDR) procedure was performed at a *q* value of 0.05 to adjust for multiple comparisons [26]. When the same threshold was applied to the matrices of the three groups, the resulting graphs would be composed of different numbers of edges. Thus, the between-group differences in network parameters would not reflect the alterations of topological organizations precisely. To control this effect, the correlation matrix of each group was converted to a binary graph with the same number of edges [25, 27] or a fixed sparsity (*S*) defined as the number of edges in a graph divided by the maximum possible number of edges of the graph [28]. Because there is no gold standard to select a single threshold, we thresholded each correlation matrix repeatedly over a wide range of sparsity (8% ⩽ *S* ⩽ 36%) and calculated the parameters of the resulting graphs with different thresholds.

### 2.6. Small-World Analysis

Small-world measures of the functional connectivity of nervous systems involve clustering coefficients, *C*_*p*_, and characteristic path length, *L*_*p*_, [29]. *C*_*p*_ is the averaged clustering coefficient over all the nodes in the graph. The clustering coefficient of a node is the ratio of the number of existing connections among the neighbors of the node to the number of all possible connections. *C*_*p*_ measures the extent of local efficiency of information transfer of a network [29, 30]. *L*_*p*_ is the average of the shortest path lengths between any pair of nodes in the graph [29]. *L*_*p*_ was measured in this study by using a “harmonic mean” distance between nodal pairs in order to avoid nodal pairs with no connections in the original definition [31]. *L*_*p*_ measures the global efficiency of the brain network [32].

In order to determine whether the experimental networks have small-world attributes, a comparison must be made to random networks with the same number of nodes and average degree. Random networks with a Gaussian degree distribution will have clustering coefficients given by *C*_*p*_^rand^ = 〈*k*〉/*N* (〈*k*〉 is the average degree of the network and *N* is the total number of nodes) [30]. The path lengths of a random network are given by *L*_*p*_^rand^ = ln⁡*N*/ln⁡(〈*k*〉) (〈*k*〉 is the average degree of the network and *N* is the total number of nodes) [30]. A real network is considered to have the small-world topology if it meets the criteria: *γ* = *C*_*p*_^real^/*C*_*p*_^rand^ > 1 and *λ* = *L*_*p*_^real^/*L*_*p*_^rand^ ≈ 1 [29].

### 2.7. Nodal Centrality

In this study, we considered the “betweenness centrality” of the nodes in the networks to investigate nodal characteristics. The betweenness *B*_*i*_ of a node *i* was defined as the number of shortest paths between any pair of nodes that run through node *I* [33]. We considered the normalized betweenness *b*_*i*_ = *B*_*i*_/〈*B*〉 (〈*B*〉 was the average betweenness of the network) as in [28]. The brain regions with high values of *b*_*i*_ were considered to be the hubs of the brain networks. 

## 3. Results

### 3.1. Psychophysical *De Qi* Response

**Figure 2:**
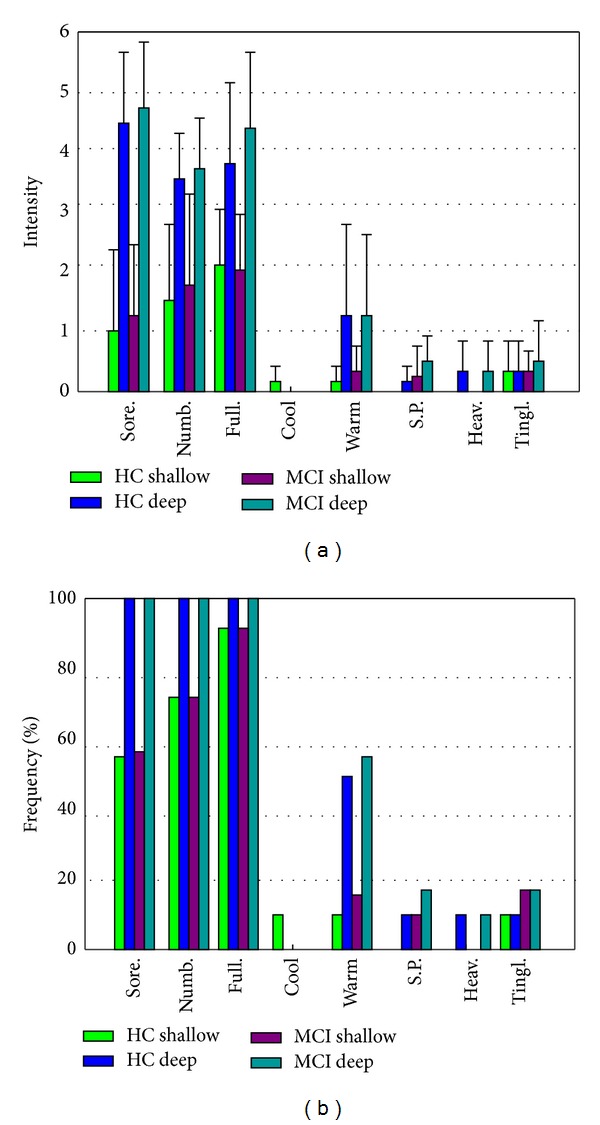
(a) The prevalence of deqi sensations. It was expressed as the percentage of the individuals in the group that reported the sensation (at least one subject experienced the seven sensations listed). (b) The intensity of sensations. It was expressed as the average score ± S.E. by measuring on a scale from 0 denoting no sensation to 10 denoting an unbearable sensation.

The prevalence of deqi sensations was expressed as the percentage of the individuals in the group that reported the sensations (Figure 2(a)). Differences did exist with respect to the type of sensations. In both the MCI and HC groups, the soreness, numbness, fullness, warmth, and heaviness were found to be more frequent for DA than that of SA. Whenever for the DA or SA condition, warmth and tingling were found to be more frequent in the MCI group than HC group. 

The intensity of sensations was expressed as the average score ± S.E. (Figure 2(b)). Differences did also exist with respect to the type of sensations. In both MCI and HC groups, the sensations of soreness, numbness, fullness and warmth were found to be stronger for DA than for SA. For both conditions, a statistical analysis found no significantly difference between the MCI and HC groups in regard to the intensity of these sensations.

### 3.2. Small-World Attributes for MCI with Different Acupuncture Needling Depth and Healthy Controls

Our results demonstrated the small-world attributes of the resting state network in the MCI patients and healthy controls. We also found that the small-world attributes also emerged during the postacupuncture resting state for both DA and SA. The *λ* and *γ* of the networks presented the function of sparsity. As the sparsity increased, the *λ* increased while the *γ* for all the networks fell down. Different from the matched random networks, all of the networks for the MCI and healthy controls demonstrated small-world architectures as they all had almost identical characteristic path lengths (*λ* ≈ 1) but were more locally clustered (*γ* > 1) over a wide range of sparsity (8% ≤ *S* ≤ 36%). At the low level of the sparsity, the *γ* of MCI networks was the largest in the three groups, while the *λ* of MCI networks was the smallest. Both measures of DA and SA for MCI patients' networks were intermediate between the MCI and healthy control groups. Notably, the small world attributes for DA in MCI patients were almost relatively similar to the healthy controls.

**Figure 3:**
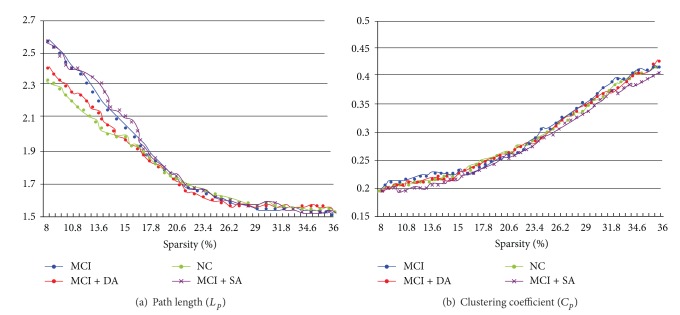
Characteristic path lengths and clustering coefficients of the whole-brain networks in MCI, DA for MCI, SA for MCI as well as healthy control subjects.

To further compare the nature of small-world attributes for MCI and healthy controls as well as DA and SA intervention effects on MCI patients, we evaluate clustering coefficients, *C*_*p*_, and characteristic path lengths, *L*_*p*_ (Figure 3). We found that the *L*_*p*_ decreased while the *C*_*p*_ increased as a function of sparsity in all the groups. Both *L*_*p*_ and *C*_*p*_ in the MCI networks were the greatest. Additionally, these values of DA for MCI networks were intermediate between MCI and healthy controls. Our findings that the *L*_*p*_ increased in DA for MCI compared with MCI suggested that deep acupuncture enhanced the compensatory of the loss in small-world attributes for treatment effects.

### 3.3. Nodal Characteristics Changes for MCI versus Healthy Control and MCI with Different Needling Depths

The changes of the betweenness centrality between MCI and healthy control, SA for MCI and MCI, as well as DA for MCI and MCI were evaluated. Compared with healthy controls, MCI patients showed centrality decreases in the brain areas of the precuneus and posterior cingulate cortex (PreCN/PCC), fusiform gyrus (FG), hippocampus, superior parietal cortex, and angular gyrus, while the centrality increases were in the brain areas of the superior frontal gyrus, ventral medial PFC (vmPFC), and lateral prefrontal cortex (LPFC) (Table 2). Compared with MCI patients, acupuncture with deep needling for MCI patients showed the centrality increased mainly in the PreCN/PCC, hippocampus, postcentral cortex as well as the anterior cingulate cortex (ACC). Compared with MCI patients, acupuncture with superficial needling for MCI patients presented the increased centrality mainly in the premotor cortex and postcentral cortex. 

## 4. Discussion

After search on the Pubmed, our study is the first report to illustrate that acupuncture with varying needling depths can induce distinct *De Qi* sensations accompanied by different modulatory effect on the reorganization of whole brain networks for MCI. The main findings of the present study were listed as follows: (i) acupuncture with deep needling can induce much stronger and a wide range of *De Qi *sensations both in intensity and prevalence, (ii) MCI patients exhibited losses of small-world attributes indicated by longer characteristic path lengths and larger clustering coefficients, compared with healthy controls, (iii) acupuncture with deep needling can exert modulatory effect to compensate the losses of small-world attributes existed in MCI patients while acupuncture with superficial needling did not, (iv) acupuncture with deep needling can enhance the nodal centrality primarily in the PreCN/PCC, hippocampus, postcentral cortex, and anterior cingulated cortex. Most of these regions present decreased nodal centrality in MCI patients. By contrast, acupuncture with superficial needling just enhanced the nodal centrality in sensory-related cortex.

In clinical settings, acupuncturists focused on “*De-Qi*” feeling during the needling treatment. This sensation was generally experienced by the patients and also by manipulating feeling of the acupuncturist when it reaches the level of “*Qi*” in the body. *De Qi* has recently drawn the attentions of many scientific researchers, and some studies propose that no appreciable therapeutic effect is obtained under a certain stimulation level, which is determined by the appearance of a particular sensation known as *De Qi* [34, 35]. In the present study, we observed that acupuncture with deep needling can induce much stronger and a wide range of *De Qi* sensations both in intensity and prevalence of needling subjective sensations. Our finding is consistent with previous report that the intensity of *De Qi* plays a key role in the clinical efficacy underlying acupuncture. Furthermore, acupuncture with superficial needling depth cannot exert promising modulatory effects on stroke recovery and generally produced weaker subjective needling sensations. This preliminary evidence may provide solid clue to demonstrate that the beneficial effects of acupuncture relied on the accurate needling depths in order to induce sufficient individual feelings. Along the same lines, acupuncture-induced sensations were mainly generated from muscle and the activity of polymodal-type receptors in deep tissues may play an important role [36].

In comparison with MCI and healthy controls, we found that MCI has some losses in the attributes of small-word brain networks and the decreased node centrality primarily in the PreCN/PCC, FG, hippocampus, superior parietal cortex, and angular gyrus. The PreCN/PCC is the site of early metabolic abnormalities in MCI [37]. As the hub of the DMN, its altered resting-state activity seems to be a meaningful functional hallmark to distinguish aMCI from normal controls. In addition, we observed that there is coexistence of decreased node centrality in the PreCN/PCC and hippocampus. This result is consistent with previous studies that damaged connectivity between the medial temporal lobe and PreCN/PCC, as a result of MTL lesions, is thought to be responsible for PreCN/PCC abnormalities [38]. For comparison between before and after acupuncture intervention, results from acupuncture with deep needling for MCI patients showed the centrality increased mainly in the PreCN/PCC, hippocampus, postcentral cortex as well as the ACC. The results indicated that acupuncture has a promising beneficial effects on MCI recovery by enhancing the nodal centrality specially in the abnormal regions related to the disease in MCI patients, aiming to rehabilitate or participant in enhancing the function of the abnormal regions. Moreover, we also noted that acupuncture with needling depths can also enhance the nodal centrality of brain regions in the ACC which cannot show abnormal functional activity in the MCI patients. One explanation can provide a possible answer that such increased nodal centrality may be a compensatory mechanism underlying MCI recovery. This inference is consistent with previous studies that an increased recruitment of the frontal is observed to compensate in AD patients in memory task [39, 40]. 

The present study has some limitations. Firstly, we cannot choose the nonspecific acupoint as controls in the design paradigm. Selection of the study design for both clinical and study investigations is prerequisites to answering the research question of interest whether acupuncture really works compared with control group. In the present study, our aim focuses on the comparison of acupuncture effects on the MCI recovery by designing different needling depths, which primarily explore the different needling individual sensations by varying needling depths. Considering that it is still not known which aspects of the acupuncture treatment, such as the mode of stimulation or location of the acupuncture point, are specific to produce these physiological effects. Using multiple controls at once is an optimal choice to unveil the relative functional specificity of acupuncture as well as effectively control the intervention of subjective placebo effects. Secondly, we instructed all the participants to keep their eyes closed during the scanning run. However, one recent research from Van Dijk et al. has demonstrated that functional connectivity strength of brain network is influenced by tasks such that fixation and eyes open rest yielded stronger correlations in the networks examined against eyes closed rest or a continuous semantic classification task [41]. Therefore, instructing subjects simply to keep their eyes open is a practical condition in studies. 

## Figures and Tables

**Table 1:** Subject characteristics.

	Patients	Controls
*N*	12	12
Age (mean ± SD)	59.3 ± 3.3	60.6 ± 5.8
Sex (M/F)	1/11	4/8
Education (year)	2.3 ± 0.4	2.4 ± 0.5
MMSE score*	26.4 ± 0.9	29.8 ± 0.4
CDR	0.5	0

Education level was determined on a discrete scale with 3 levels: low = 1, middle = 2, and high = 3. Data are presented as mean ± SD. MMSE: mini-mental state examination. CDR: clinical dementia rating. *Statistically significant difference at the *P* < 0.0001 level.

**Table 2:** Brain areas showing significant difference in nodal centrality.

A. Nodal centrality changes for MCI versus healthy controls		
Regions	Normalized betweenness, b_i_	
	MCI	Healthy controls
*Decreased nodal centrality *		
PreCN/PCC	0.9536	3.5475
Fusiform gyrus	0.2843	1.7431
Hippocampus	0.3302	1.8622
Superior parietal cortex	0.8539	2.9568
Angular gyrus	0.6735	1.7569
*Increased nodal centrality *		
Superior frontal gyrus	4.0174	0.1526
Ventral medial PFC	4.1528	0.1947
Lateral prefrontal cortex	3.9524	0.1751

B. Nodal centrality changes for MCI DA versus MCI		
*Increased nodal centrality *		
PreCN/PCC	2.1768	0.9536
Hippocampus	1.9244	0.3302
Postcentral cortex	2.1192	0.7852
Anterior cingulate cortex	3.1568	1.4679

C. Nodal centrality changes for MCI SA versus MCI		
*Increased nodal centrality *		
Premotor cortex	3.1894	1.1295
Postcentral cortex	2.7625	0.7852

Abbreviations: PreCN/PCC: precuneus and posterior cingulate cortex; PFC: prefrontal cortex.
